# Metabolomic profiling of exhaled breath condensate for the diagnosis of pulmonary aspergillosis

**DOI:** 10.3389/fcimb.2022.1008924

**Published:** 2022-09-08

**Authors:** Shuo Wei, Yi-sheng Chen, Yi Shi

**Affiliations:** ^1^ Department of Infectious Disease, Shengli Clinical Medical College of Fujian Medical University, Fujian Provincial Hospital, Fuzhou, China; ^2^ Department of Clinical Laboratory, Shengli Clinical Medical College of Fujian Medical University, Fujian Provincial Hospital, Fuzhou, China; ^3^ Department of Respiratory Medicine, Jinling Hospital, Nanjing, China

**Keywords:** pulmonary aspergillosis, exhaled breath condensate, ultra high-performance liquid chromatography, high-resolution mass spectrometry, diagnosis

## Abstract

**Objective:**

This study aims to ascertain the unique metabolic profile of exhaled breath condensate (EBC) samples in pulmonary aspergillosis (PA) patients, and explore their usefulness for the diagnosis of PA.

**Methods:**

A total of 133 patients were included in the study, including 66 PA patients (invasive pulmonary aspergillosis, n=3; chronic pulmonary aspergillosis, n=60; allergic bronchopulmonary aspergillosis, n=3) and controls (n=67). Ultra high-performance liquid chromatography coupled with high-resolution mass spectrometry(UHPLC-HRMS) was used to analyze EBC samples. Metabolic profiling of EBC samples that were collected from 22 CPA patients at various times during treatment (before treatment, <1 month, 1–2 months, 2–3 months, 3–6 months, and ≥6 months after treatment initiation) were performed using UHPLC-HRMS. Potential biomarkers were evaluated using cluster analysis, Venn diagram and receiver operating characteristic analysis (ROC).

**Results:**

A total of 47 metabolites of potential interest were detected in the EBC samples. Further investigation showed that Asperpyrone C, Kotanin, Terphenyllin, Terrelumamide B, and Cyclotryprostatin D could be used as a diagnostic biomarker for PA. The classification between metabolic profiling of EBC samples from PA patients and controls was good with a sensitivity of 100%, specificity 89.6% for patients with PA, respectively. Venn diagram analysis of these biomarker candidates displayed three main types of compounds, which could be used for the further discrimination of aspergilloma and chronic cavitary PA. In addition, antifungal treatment had a limited influence on the value of the EBC results.

**Conclusions:**

This metabolomic approach using UHPLC-HRMS could be used as a noninvasive method for the diagnosis of PA.

## Background

The diagnosis of pulmonary aspergillosis (PA) remains a challenge. Currently, diagnosis is mainly based on routine testing, such as pathological evidence,culture and direct microscopic examination. However, these routine methods have poor sensitivities (Patterson et al., 2016). Although bronchoalveolar lavage fluid (BALF) has been applied to galactomannan (GM) which is a component of the cell wall of *Aspergillus* assay and quantitative polymerase chain reaction (PCR) for diagnosis of PA and some progress has been made (Johnson et al., 2014; Patterson et al., 2016; Ullmann et al., 2018; Donnelly et al., 2020), the bronchoscopy examination is an invasive procedure and unsuitable for repeated use in practice.

Although exhaled breath analysis of volatile organic compounds (VOCs) by electronic nose (eNose) technology has been evaluated for the diagnosis of invasive aspergillosis and showed a good performance where the eNose discriminated *Aspergillus fumigatus* from bacteria/yeasts, or *Rhizopus arrhizus* with an accuracy of 92.9% and 100%, respectively (de Heer et al., 2016); However, this method requires special expensive equipment and is not available in most centers. In another study conducted in 2014, VOCs in exhaled breath was analyzed using gas chromatography mass spectrometry and the VOCs could be used to differentiate invasive pulmonary aspergillosis (IPA) from other pneumonias (Koo et al., 2014). Unfortunately, this study had several disadvantages, such as inconvenient sample preparation, expensive machines, and only invasive PA was included not chronic pulmonary aspergillosis (CPA). Recently, several studies have reviewed the application of exhaled breath condensate (EBC) samples in the assessment of pulmonary diseases, such as asthma, obstructive sleep apnea, and malignant pleural mesothelioma (De Luca Canto et al., 2015; Horváth et al., 2017; Peel et al., 2017; Töreyin et al., 2020).

To date, few studies have been conducted to investigate the application of EBC samples in the diagnosis of PA. In 2018 a study reported that GM was detectable in EBC for the diagnosis of IPA in immunocompromised patients, however, this study did not include CPA patients (Bhimji et al., 2017).Therefore, this prospective study aims to ascertain the unique metabolic profile of EBC samples in PA patients and explore their usefulness for the diagnosis of PA.

## Methods

### Ethics

The study was conducted at Fujian Provincial Hospital in Southeast China. This study was approved by the Ethics Committees of Fujian Provincial Hospital (Ethical approval number K2021-03-041). Written informed consent was obtained from all suspected patients before the study started.

### Patients

**Figure 1:**
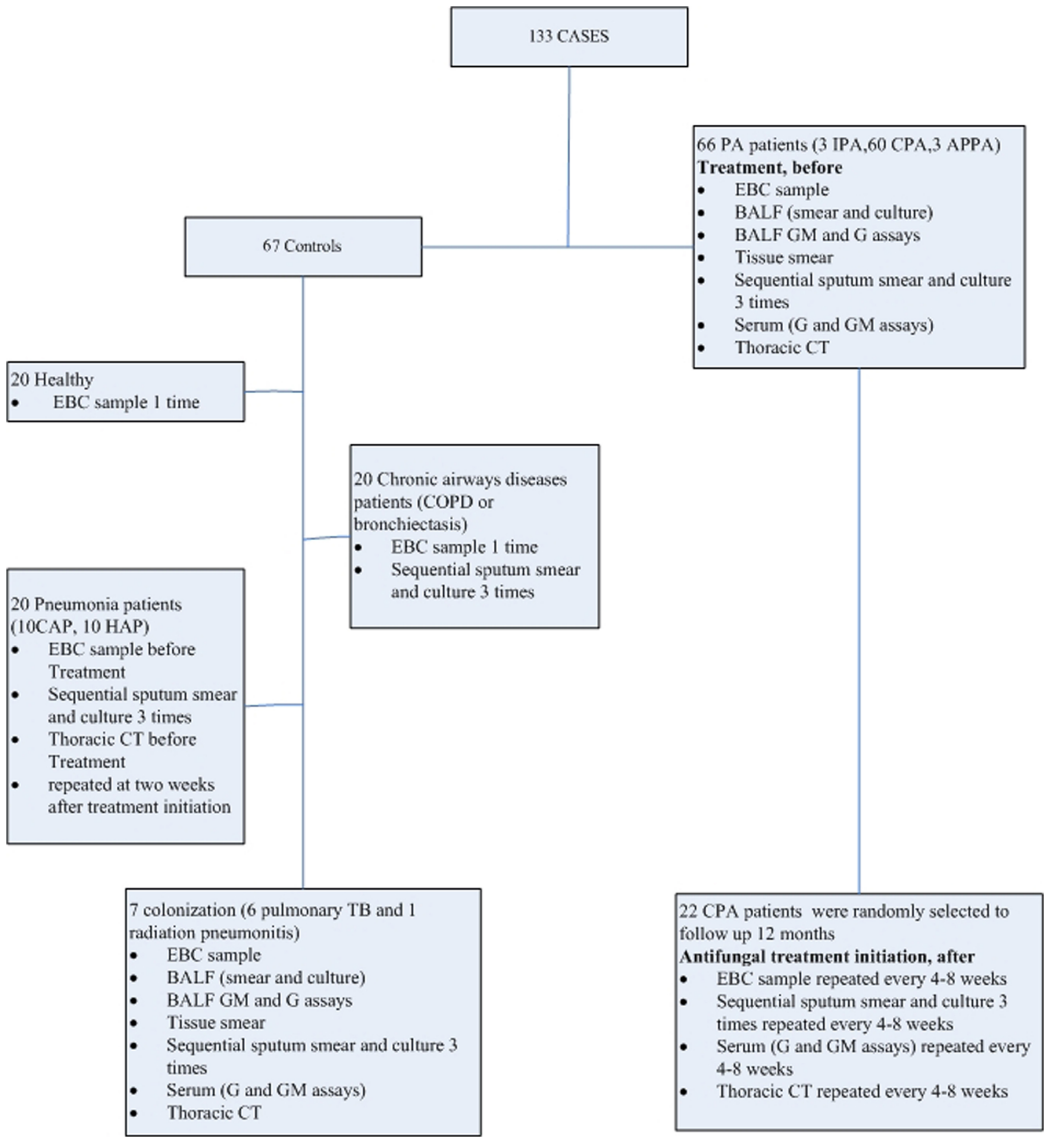
Flow chart of study protocol.

Between January 2018 and November 2019, PA patients who admitted to the center were included for further analysis, including IPA, Allergic Bronchopulmonary Aspergillosis (ABPA), and CPA [Simple aspergilloma, Aspergillus nodule, Chronic cavitary pulmonary aspergillosis (CCPA), Chronic fibrosing pulmonary aspergillosis (CFPA), Subacute invasive pulmonary aspergillosis formerly called chronic necrotizing pulmonary aspergillosis (CNPA)]. Controls were also set, including pneumonia (community acquired pneumonia (CAP), hospital acquired pneumonia (HAP)), chronic respiratory tract infection (such as COPD and bronchiectasis), and healthy volunteers. In addition, *Aspergillus* respiratory tract colonization control was also set. Then, clinical specimens, such as serum, sputum, BALF, and lung tissues, were collected from patients or healthy volunteers. Routine assays, such as culture, smear, GM, and 1-3-β-D glucan assays, metagenomic next-generation sequencing (mNGS), and histological examinations, were performed at various times during treatment (before treatment, <1 month, 1–2 months, 2–3 months, 3–6 months, and ≥6 months after treatment initiation), and EBC samples were collected in parallel to determine metabolic profiles for comparison. According to the 2016 consensus reached by the IDSA of PA, PA patients received voriconazole (6 mg/kg IV every 12 h for 1 d, followed by 4 mg/kg IV every 12 h; oral therapy can be used at 200–300 mg every 12 h or weight based dosing on a mg/kg basis) (Patterson et al., 2016). The detailed study protocol is shown in **Figure 1**.

The diagnostic criteria for patients were described briefly, as follows: proven or probable IPA were defined according to the revision and update of the European Organization for Research and Treatment of Cancer and the Mycoses Study Group Education and Research Consortium (Donnelly et al., 2020). The criteria for ABPA diagnosis was adapted from a review published in “*The Journal of Allergy and Clinical Immunology*” (2012) (Knutsen et al., 2012). The diagnosis of CPA requires a combination of characteristics: one or more cavities with or without a fungal ball present or nodules on thoracic imaging, direct evidence of *Aspergillus* infection (microscopy or culture from biopsy) or an immunological response to *Aspergillus* spp. and exclusion of alternative diagnoses, all present for at least 3 months (Denning et al., 2015). In addition, CPA was further classified as: Simple aspergilloma, Aspergillus nodule, CCPA, CFPA, CNPA (Denning et al., 2015). Colonization with *Aspergillus* spp. in respiratory samples was defined based on: without any evidence for *Aspergillus*- associated infection, and one or more criteria necessary for a diagnosis of putative PA are absent. Patients were excluded due to unavailable EBC samples and requirement of mechanical ventilation.

### EBC samples

At the same site, EBC was collected using a commercially available condenser (RTube, Respiratory Research, Charlottesville, Virginia, USA). No fasting required before sampling. It is a portable Collection Devices for the Study of Respiratory Droplets in Exhaled Breath Condensate. Large “Tee” section separates saliva from the exhaled breath and prevents it from entering the condensation tube Custom duckbill valve/nozzle enhances condensation efficiency and produces high condensate volumes. Exhaled Breath Condensate (EBC) is composed of droplets of Airway Lining Fluid (ALF) evolved by turbulence from all lung compartments and held in a matrix of condensed moisture from the breath. These droplets contain numerous biomarkers including DNA, RNA, mRNA, proteins, metabolites, and volatile organic compounds (VOC). Typical condensate fluid yield is 200 microliters/minute for an adult at normal tidal breathing effort. 7-10 minute collection time is commonly employed. EBC Volume of Condensate Collected at least 1000 microliters. Prepare the cooling sleeve before taking samples. The cooling sleeve is necessary for efficient condensate collection. Usually stored in a laboratory freezer Temperature(-80°) until needed. After sample collection has been completed and the sample stored properly and place the cooling sleeve back into the protective bag and refreeze. This unique feature allows for easy integration of the RTube into existing studies and allows large amounts of EBC data to be collected with ease from subjects in the clinic, hospital, home, workplace, school, or any other reasonable environment. Then, EBC samples were preserved in aliquots and stored at −80°C until analysis. Ultra high-pressure liquid chromatography (UHPLC) and electrospray ionization (ESI) coupled with high-resolution mass spectrometry (HRMS) (UHPLC/ESI–HRMS) analyses were performed using a Waters Acquity Ultra-Performance Liquid Chromatography (LC) (UPLC) system (Waters, Milford, MA, USA) and Waters Xevo G2-XS Q-TOF system, as outlined in the **Supplementary Methods**. (**Supplementary Material 1**). After removing background noise, all compounds identified with a mass spectrometry response value of > e^4^ were selected for further analysis. Then, a comparison between PA patients and healthy volunteers was performed to identify potential compounds for the diagnosis of PA. In addition, based on the existing literature, a dataset of the metabolites of *Aspergillus* sp. was manufactured and then differential compounds found in the EBC samples were determined if they had the same m/z value (differences <0.05 Da) was matched. The investigators that performed the metabolite identification were blinded to clinical data.

Venn diagram analysis of the EBC metabolites that were identified and cluster analysis of PA patients was then performed. The diagnostic performance of the EBC method was evaluated and compared with routine methods, or BALF GM assay combined with mNGS. In addition, the potential of the EBC method during antifungal treatment was investigated.

### Statistical analysis

Statistical analyses were performed by SPSS 23.0. Venn diagrams (OriginPro 2019, version 9.6.0.172; OriginLab) and cluster analyses were used to screen out the differential metabolomic profiling of EBC between patients with different aspergillosis. Then, clinical characteristics aspects were compared between PA patients and controls and among PA subgroups. Continuous variables were presented with mean ± standard deviation and compared with ANOVA. Categorical variables were presented with percentages (frequency) and compared with Chi-squared (χ^2^) or Fisher’s exact tests. Quantitative analysis was performed by UHPLC/ESI–HRMS, sensitivity and specificity were calculated, respectively. The receiver operating characteristic (ROC) analysis was used to compare different diagnostic methods, and the agreement between them was assessed using Cohen’s kappa coefficient. A two-sided *p-*value of <0.05 was considered significant.

## Results

### Patients

During the study, a total of 66 PA patients (IPA, n=3; CPA, n=60; ABPA, n=3) were included. Then, twenty two of CPA cases were selected randomly and followed-up for 1 year. Controls (n=67) that included pneumonia (n=20; CAP), n=10; HAP, n=10), chronic airway disease (n=20), *Aspergillus* respiratory tract colonization (n=7; pulmonary tuberculosis, n=6; radiation pneumonitis, n=1), and healthy volunteers (n=20) were included. No significant differences existed between the five groups (PA, colonization, pneumonia, chronic airway disease, and healthy) in clinical characteristics aspects included number, age, gender, risk factor, and APACHEII (**Table 1**).

**Table 1:** Clinical characteristics of subjects included in this study.

Variables	PA	Aspergillus colonization ^a^	Pneumonia^c^	Chronic airway disease^d^	Healthy	*p*-value
Number	66	7	20	20	20	
Age (years)	56.86 ± 11.08	62.86 ± 8.80	57.20 ± 10.40	60.35 ± 9.64	58.65 ± 10.79	0.500
Gender [male (%)]	38 (57.6)	3 (42.9)	10 (50.0)	12 (60.0)	10 (50.0)	0.888
^b^Risk factor [(n) %]	5 (7.6)	1 (14.3)	1 (5.0)	1 (5.0)	0 (0.0)	0.771
^e^APACHEII	6 (4.75,7)	8 (3,9)	6 (4.25,8)	7 (5,7.75)	N/A	0.204

a colonization (6 pulmonary TB and 1 radiation pneumonitis).

b The risk factors for PA include prolonged neutropenia, hematologic malignancy, allogeneic HSCT recipients, solid organ transplant (SOT) recipients, corticosteroid use, T or B-cell immunosuppressants use, inherited severe immunodeficiency, and acute graft-versus-host disease grade III or IV (Donnelly et al., 2020).

c Sputum culture for pneumonia (n=20): Streptococcus pneumoniae pneumonia (n = 1), Stenotrophomonas maltophilia (n = 1), Pseudomonas aeruginosa (n = 1), methicillin resistant Staphylococcus aureus (n = 1), and others (-).

d Chronic respiratory tract infection: Stenotrophomonas maltophilia (n=1), Pseudomonas aeruginosa (n=1), and others (-).

e APACHE II, acute physiology and chronic health evaluation scoring system; Healthy group, N/A.

### Comparison between healthy and PA groups

**Figure 2:**
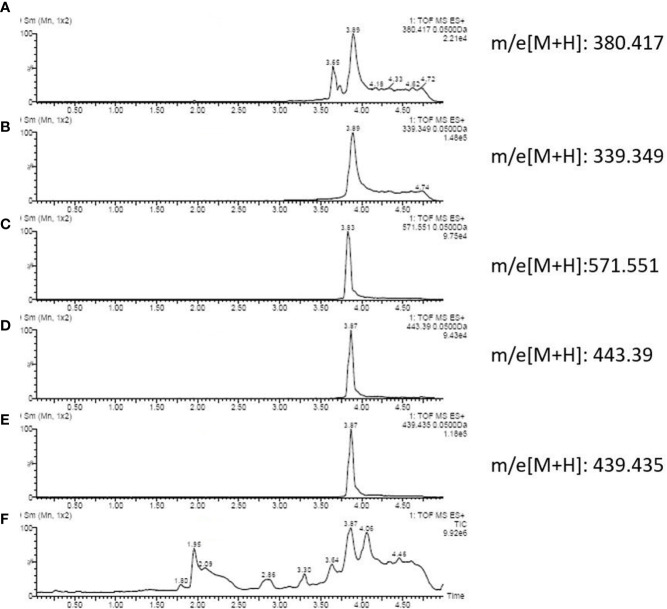
Ion chromatography of an EBC sample that consists of: **(A)** Cyclotryprostatin D; **(B)** Terphenyllin; **(C)** Asperpyrone C; **(D)** Terrelumamide B; **(E)** Kotanin; and **(F)** total metabolites.

**Figure 3:**
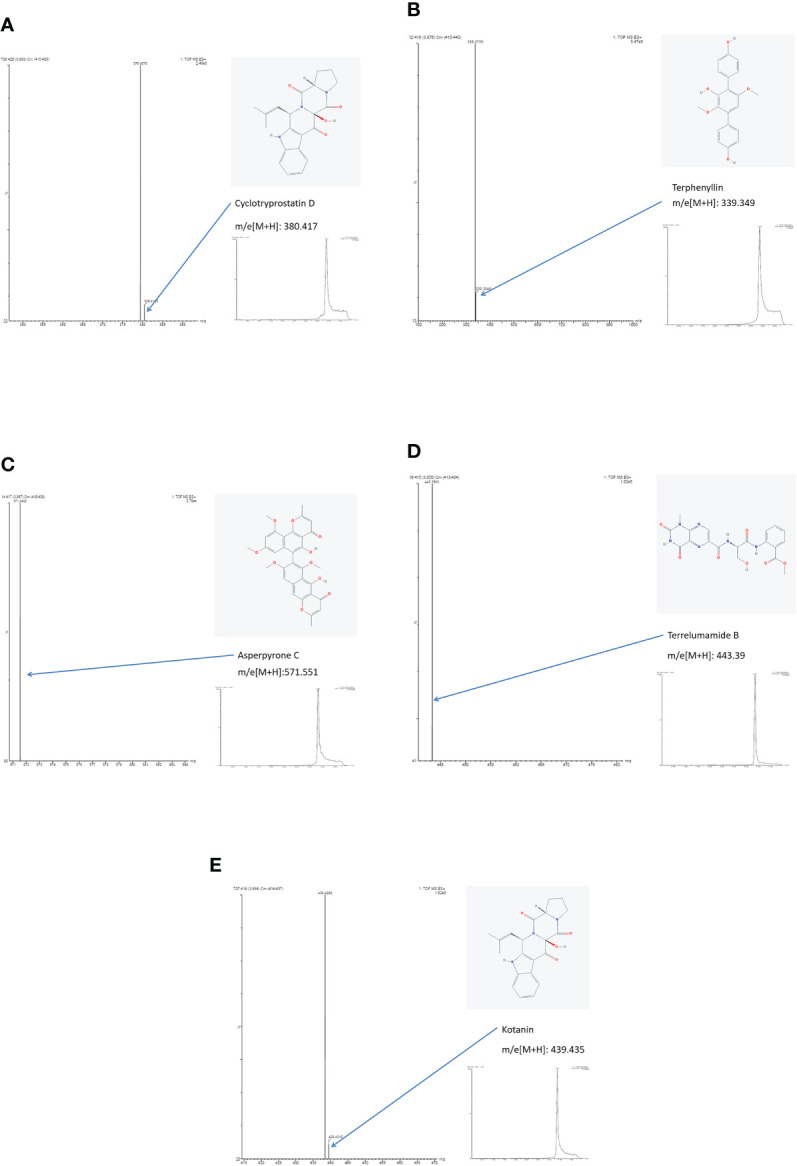
Results of ion chromatography and mass spectrometry: **(A)** Cyclotryprostatin D; **(B)** Terphenyllin; **(C)** Asperpyrone C; **(D)** Terrelumamide B; **(E)** Kotanin.

By literature review, a data set of primary *Aspergillus* metabolites and secondary metabolites were constructed with at least 500 compounds for the identification of candidates (**Supplementary Material 2**). A comparison of UHPLC/ESI–HRMS results between healthy (n = 20) and PA (n = 66) groups was performed. Approximately 47 metabolites were different between groups which were selected for further analysis. Among the PA group, 10 of the 47 metabolites were positive in >80% of cases (**Table 2**). Owing to the high analyte sensitivity of UHPLC/ESI–HRMS revealed by ion responses and chromatographic peak shape, five metabolites were excluded and the remaining five were included, as follows: Asperpyrone C, Terphenyllin, Kotanin, Terrelumamide B, and Cyclotryprostatin D. Asperpyrone C was detected in all cases with PA, UHPLC/ESI–HRMS analysis revealed that Terphenyllin was more abundant compared with the others, and all five metabolites previously mentioned could easily be identified (**Figures 2**, **3**). In addition, the five metabolites were absent in 40 patients with pneumonia and chronic respiratory tract infection. However, one or more metabolite of the five metabolites mentioned above could be detected in the EBC samples among the seven cases with *Aspergillus* colonization.

**Table 2:** Positive rates for 47 metabolites identified in EBC samples among patients with PA.

No	Compound	m/z value	Positive rate (%)
1	2-hydroxy-3-methyl-1,4-benzoquinone, C7H6O3	138.121	6.1
2	Penicillic acid, C8H10O4	170.163	83.3
3	Naphthalic anhydride, C12H6O3	198.174	93.9
4	Aspergillomarasmine B, C9H14N2O8	278.216	3.0
5	aflatoxicol (Aflatoxin Ro), C17H14O6	314.289	22.7
6	3-hydroxyterphenyllin, C20H18O6	354.353	92.4
7	JBIR-138, C19H23O7	363.382	3.0
8	Demethylasterriquinone, C24H20N2O5	416.426	13.6
9	Arugosin E, C25H26O6	422.470	4.5
10	Kotanin, C24H22O8	438.427	97.0
11	Terrelumamide B, C19H18O7N6	442.382	81.8
12	Terrelumamide A, C20H20O7N6	456.409	42.4
13	territrem A, C28H30O9	510.532	4.5
14	Dianhydro-aurasperone C, C31H24O10	556.516	19.7
15	Asperpyrone C, C32H26O10	570.543	100.0
16	Emestrin, C27H22N2O10S2	598.601	4.5
17	Flufuran, C6H6O4	142.109	22.7
18	Terphenyllin, C20H18O5	338.354	83.3
19	Asterriquinone (1), C25H22N2O5	430.453	16.7
20	Fumitremorgin C/Tryptoquivaline C, C29H30N4O7	546.571	13.6
21	Nigerapyrone B, C21H20O3	320.382	81.8
22	Cyclotryprostatin D, C21H21N3O4	379.409	80.3
23	Candidusin A 804, C20H16O6	352.337	72.7
24	Folipastatin, C23H24O5	380.434	74.2
25	Asparvenone, C12H14O4	222.237	62.1
26	Erythroglaucin, C16H12O6	300.263	3.0
27	versiconal acetate, C20H16O9	400.336	1.5
28	Pseurotin B, C22H25NO9	447.435	22.7
29	cis-4-Hydroxymellein, C10H10O4	194.184	1.5
30	phthalide or chromanol (3), C11H12O5	224.210	1.5
31	Bianthrone and secoanthraquinone secondary metabolite 1, C16H14O7	318.278	4.5
32	phthalide or chromanol (7), C16H20O7	324.326	4.5
33	Phenylahistin, C20H22N4O2	350.414	4.5
34	Sydoxanthone C, C17H14O7S	362.354	4.5
35	5,6-dimethoxysterigmatocystin, C20H16O8	384.336	4.5
36	Kipukasin H, C18H20N2O9	408.359	7.6
37	Butyrolactone I, C19H16O7	356.326	7.6
38	Emerixanthone D, C27H30O8	482.522	9.1
39	Phomaligin A, C16H25NO5	311.373	28.8
40	brevianamide M, C18H15N3O3	321.330	3.0
41	Viriditoxin, C34H30O14	662.594	37.9
42	Neosartorin, C34H32O15	680.609	13.6
43	Cryptoechinuline E, C20H19N3O3	349.383	4.5
44	Xanthoascin (2), C22H16N2O4	372.373	10.6
45	tryptoquivaline E, C22H18N4O5	418.402	3.0
46	Fumiquinazoline K, C26H23N5O4	469.492	6.1
47	Fumiquinazoline C, C24H21N5O4	443.455	3.0

### Hierarchical cluster analysis

**Figure 4:**
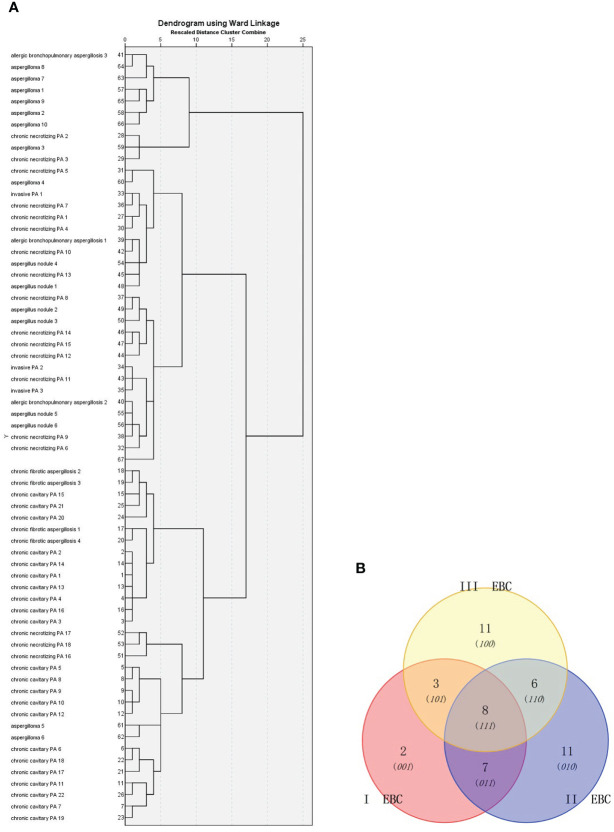
Cluster analysis of: **(A)** 66 PA patients based on Ward chain; and **(B)** Venn diagram analysis of the EBC metabolites identified from these patients. I EBC : **Table 2** (26, 27). II EBC: **Table 2** (29-35, 38,40- 42). III EBC: **Table 2** (1, 4, 7, 9,13,16, 43-47).

First, PA patients were divided into seven groups: invasive PA, Aspergilloma, Aspergillus nodule, chronic cavitary PA, chronic fibrosis PA, chronic necrotizing PA, and allergic bronchopulmonary aspergillosis. Then, a Venn diagram was constructed for the 47 metabolites to show the similarities between the EBC signature for the metabolic panel. No significant difference in the clinical characteristics was observed between the seven groups (**Table 3**, *p*>0.05). The cluster analysis identified three groups: (1) chronic cavitary PA, chronic fibrotic aspergillosis; (2) invasive PA, allergic bronchopulmonary aspergillosis, aspergillus nodule, chronic necrotizing PA; and (3) aspergilloma (**Figure 4A**). The Venn diagram identified three main types of metabolites that partially overlapped (**Figure 4B**).

**Table 3:** Clinical characteristics of patients between seven groups.

	IPA	Aspergillus globules	Aspergillus nodule	chronic cavitary PA	chronic fibrosis PA	chronic necrotizing PA	allergic bronchopulmonary aspergillosis	*p*-value
Number (n)	3	10	6	22	4	18	3	
Age (years, mean ± SD	58.00 ± 5.00	57.90 ± 10.91	52.17 ± 13.24	57.55 ± 12. 13	57.50 ± 14.34	56.17 ± 9.33	60.00 ± 17.35	0.340
Gender [n (%)]								0.987
Male	2 (66.7)	6 (60.0)	4 (66.7)	13 (59.1)	2 (50.0)	9 (50.0)	2 (66.7)	
^a^Risk factors [n (%)]	1 (33.3)	0 (0.0)	0 (0.0)	2 (9.1)	0 (0.0)	1 (5.6)	1 (33.3)	0.277
APACHE II	9 (6),	4 (3.75,6.5)	4.5 (3,6.75)	5 (4,6.25)	6.5 (5.25,7.75)	7 (4.75,9)	6 (6),	0.097
Smear (sputum or BALF)	1 (33.3)	1 (10.0)	1 (16.7)	3 (13.6)	1 (25.0)	2 (11.1)	0 (0)	0.969
Culture (sputum or BALF)	2 (66.7)	3 (30.0)	2 (33.3)	8 (36.3)	2 (50.0)	5 (27.8)	2 (66.7)	0.787
BALF GM≥1.0	1 (33.3)	4 (40.0)	3 (50.0)	15 (68.2)	3 (75.0)	11 (61.1)	2 (66.7)	0.741

a Risk factors for PA include prolonged neutropenia, hematologic malignancy, allogeneic HSCT recipients, solid organ transplant (SOT) recipients, corticosteroid use, T or B-cell immunosuppressants use, inherited severe immunodeficiency, and acute graft-versus-host disease grade III or IV (Donnelly et al., 2020). Five patients had ≥1 risk factors, including one renal transplant (n = 1) and corticosteroid use (n = 4).

Of the 66 PA patients, 40 were tested for tissue culture and pathological examination and 18 have positive results, 12 were tested for immunoCAP Aspergillus-specific IgG test and 10 have positive results, nine were tested for BALF mNGS and three have positive results, and no BALF PCR was performed.

### Diagnostic performance of metabolic panels of EBC

For the diagnosis of PA, the role of metabolic panels of EBC were assessed using Cohen’s kappa coefficient (κ), sensitivity, specificity, positive and negative predictive values and were reported as follows: 0.895, 100% (66/66), 89.6% (60/67), 90.4% (66/73), and100% (66/66), respectively (**Table 4**). Between the 133 patients included in the study, the EBC method agreed with the clinical diagnosis in most patients [n = 126 (94.7%)] and disagreements between the two were found in seven cases, which showed confirmed colonization.

**Table 4:** Performance of EBC method in the diagnosis of PA.

	PA	Controls	Total	Kappa indices	Agreement
EBC method (+)	66	7	73	0.895	94.7%
EBC method (-)	0	60	60		
Total	66	67	133		

Kappa indices, 0.895 (0.821–0.969, 126/133); Sensitivity, 100% (0.821–0.969, 66/66); Specificity, 89.6% (79.1%– 95.3%, 60/67); Negative predictive value, 100% (92.5%–100.0%, 66/66); Positive predictive value, 90.4% (80.7%–95.7%, 66/73).

**Figure 5:**
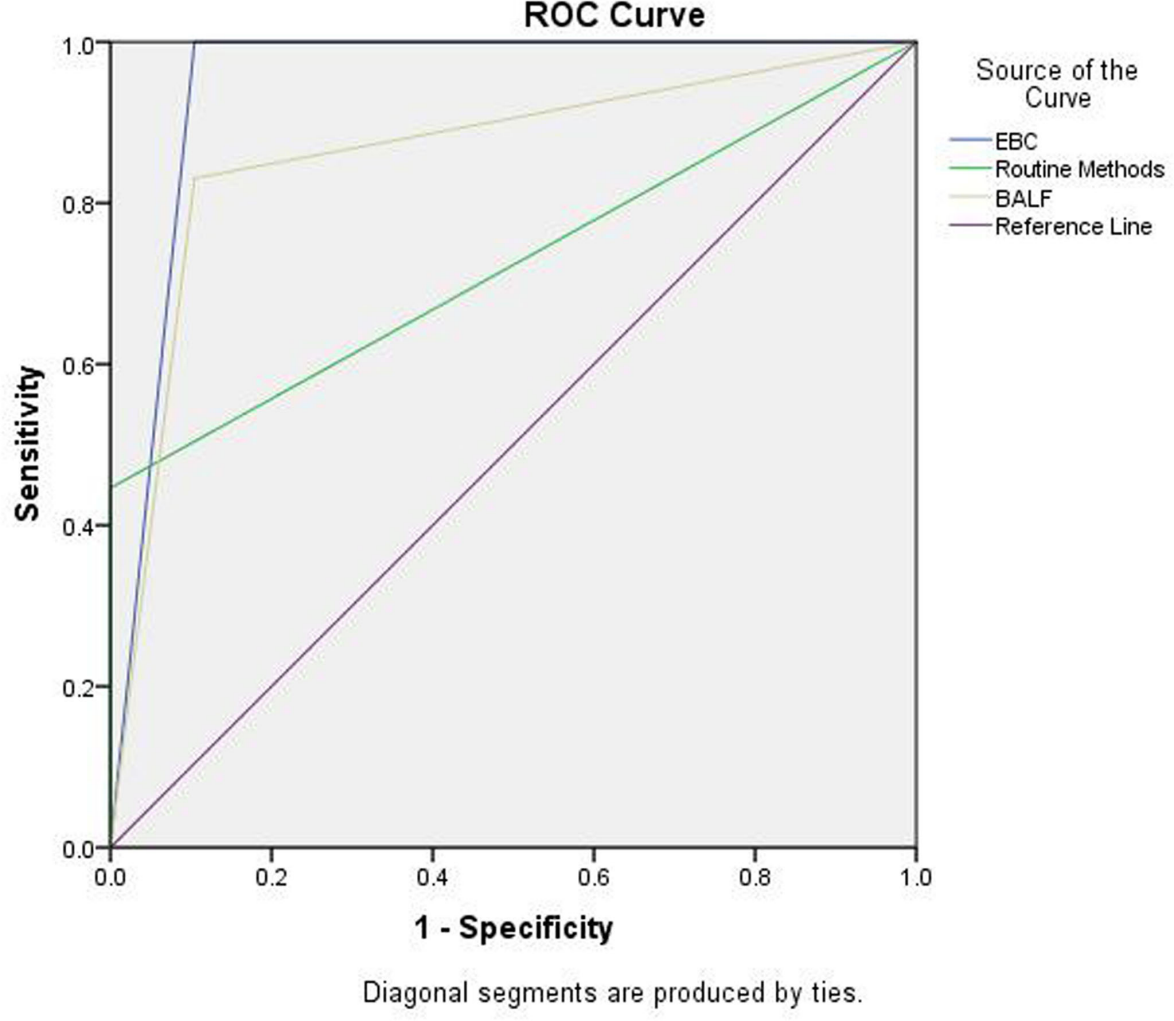
ROC analysis for EBC method, routine assays, and BALF GM + mNGS in the diagnosis of PA. Area under ROC curve: EBC, 0.948 (0.904, 0.991); routine method, 0.723 (0.634, 0.812); and BALF GM + mNGS 0.863(0.795,0.931).

In addition, ROC analysis was used to compare the performance between the EBC method and routine methods [e.g., tissue examination, culture and smear (e.g., sputum, BALF, bronchial brushing, and tissues)] or BALF GM assay combined with mNGS (BALF GM+ mNGS). The positive results of each methods: (1) EBC method: PA patients (100%, 66/66), controls, (10.4%, 7/67); (2) Combination of tissue examination and culture and smear using several specimens (e.g., sputum, bronchoalveolar lavage fluid, bronchial brushing, and tissue): PA patients (45.5%, 30/66), controls (0%, 0/67); (3) BALF GM + mNGS: PA patients (81.8%, 54/66), controls (10.4%, 7/67). The area under the ROC curve of the EBC method was 0.948 and was higher than that of the routine methods (0.723), or BALF GM assay combined with mNGS [0.863; p<0.05 (**Figure 5**)].

### Impact of antifungal therapy

**Figure 6:**
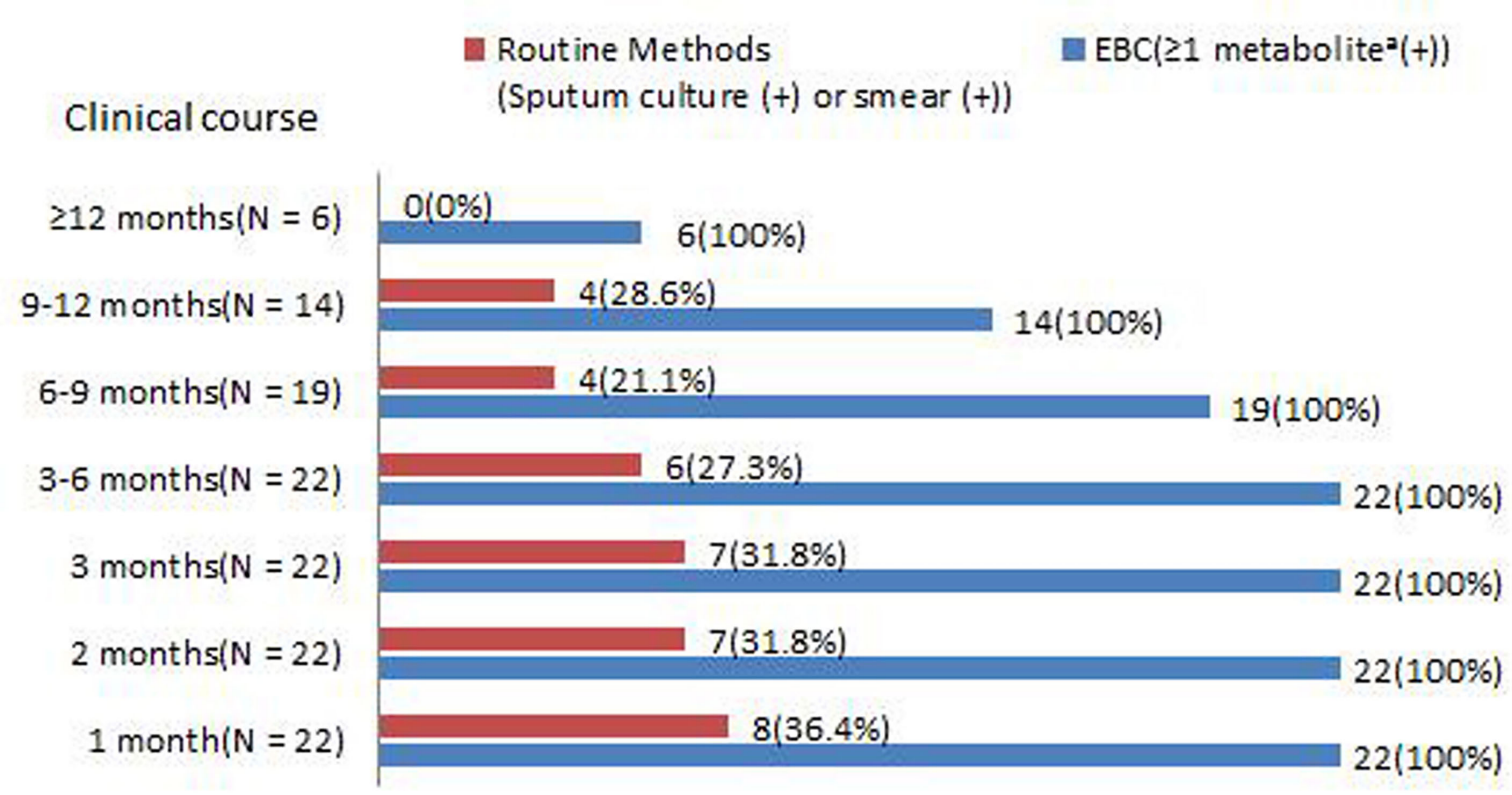
Positive rates of (sputum culture and smear) and ≥1 metabolite in EBC samples among PA patients during antifungal therapy. **^a^** Asperpyrone C, Kotanin, Terphenyllin, Terrelumamide B, and Cyclotryprostatin D.

In total, 22 CPA patients were followed-up for 1 year. Routine (sputum culture and smear) and EBC methods were tested after 1, 2 and 3 months, 3–6 months, 6–9 months, 9–12 months, and ≥12 months of antifungal therapy. The sensitivity of the EBC method was always 100% (**Figure 6**). In addition, considering corresponding conditions, patients were administrated with 6-9 months, 9-12 months, or ≥12 months antifungal therapy separately, all EBC samples were tested as negative at the therapy termination. In contrast, the sensitivity of routine methods appeared to decrease during the treatment, only 20% were positive in routine methods after 6 months of therapy and none of them were positive when the study was completed.

## Discussion

In this study, metabolites produced by *Aspergillus* sp. were exhaled and absorbed into the condensing breath. Using a condenser RTube EBC samples were collected for UHPLC/ESI– HRMS analysis and metabolites, such as Asperpyrone C, Kotanin, Terphenyllin, Terrelumamide B, and Cyclotryprostatin D5 demonstrated a significant difference between the PA group (IPA, CPA, and ABPA) and healthy volunteers, pneumonia, or chronic respiratory tract infection. Further analysis showed the metabolic panels of EBC samples had a sensitivity of 100% and specificity of 89.6% for the diagnosis of PA.

Over the last decades, several assay, such as GM assay, *Aspergillus* IgG antibodies serology test, and molecular methods (eg, PCR, and mNGS) were evaluated in the field of IPA and CPA diagnosis. According to the guidelines, mycological evidence for probable IPA includes the following items: 1) GM assays (at least one): single serum or plasma: ≥1.0; BAL fluid: ≥1.0; single serum or plasma: ≥0.7 and BAL fluid ≥0.8. 2) *Aspergillus* PCR (at least one): plasma, serum, or whole blood (+, 2 or more consecutive PCR tests); BAL fluid (+, 2 or more duplicate PCR tests); plasma, serum, or whole blood (+, at least 1) and BAL fluid (at least 1). 3) *Aspergillus* culture: sputum (+), BAL(+), bronchial brushing (+), or bronchial aspirate (+) (Donnelly et al., 2020). Besides, if patients have one or more cavities, characteristic are consistent with CPA, and other diagnosis has been excluded, a diagnosis of CPA can be confirmed when met the following items: *Aspergillus* IgG antibodies serology or precipitins (+), GM (+) or *Aspergillus* DNA (+) in respiratory fluids (Denning et al., 2015). Recently, mNGS was introduced in practice and presented as a promising diagnostic tool for the diagnosis of infectious lesions. This method detected and identified a large variety of pathogens during pulmonary infection, including *Aspergillus* sp (Huang et al., 2020).

In this study, a comparison of diagnostic performance between EBC method、BALF GM +mNGS, and routine methods were assessed. It was found that the EBC method has the highest AUC value with about 1.0 and further ROC curve analysis demonstrated that the EBC method was superior to the other two methods for the diagnosis of PA. The data suggested that the sensitivity of the EBC method was higher than that of the other two other methods and BALF GM+mNGS and EBC methods had similar specificities without significant difference. Due to the small number of IPA, and ABPA enrollment, and the fact that the diagnosis of ABPA (without routine methods and GM assay) (Knutsen et al., 2012) the diagnostic performance of IPA, CPA, and ABPA for EBC was not further analyzed separately compared with routine methods and with BALF GM + mNGS tests. It was reported that, in patients with impaired immunity of having IPA, serum GM had a sensitivity of 0.71 and specificity of 0.89. BAL GM had a sensitivity of 0.84 and specificity of 0.88. Serum or whole blood PCR had a sensitivity of 0.81 and specificity of 0.79. BAL PCR had a high sensitivity of 0.90 and specificity of 0.96 (Haydour et al., 2019). Regarding the role of GM assay in CPA, serum GM appears to have limited value for CPA diagnosis. This is because the sensitivity of serum GM was only 23% (Shin et al., 2014). In addition, the performance of GM assay for CPA diagnosis depended on the cut off value selected. For example, the sensitivity and specificity of GM assay in BAL fluid specimens was 77.2% and 77.0%, respectively (with a cut-off level of 0.4) (Izumikawa et al., 2012). In another study the BAL GM-antigen detection test had a sensitivity and specificity of 85.7% and 76.3%, respectively, with a cut-off level of >0.5 (Kono et al., 2013). In a recent study, the best cut off value for serum and BALF-GM was 0.55 (area under the ROC curve [AUROC], 0.605; sensitivity, 38%; specificity, 87%) and 1.375 (AUROC, 0.836; sensitivity, 68%; specificity, 93%), respectively. At a cutoff value of 2.5, BALF GM had a sensitivity and specificity of 50% and 100%, respectively (Sehgal et al., 2019).

Of the 66 PA patients, three groups were identified by cluster analysis: (1) CCPA,CFPA; (2) IPA, ABPA, aspergillus nodule, CNPA; and (3) aspergilloma. It is noted that according to the results previously mentioned, cluster analysis is associated with infectious severity and is not related to the immune responses to *Aspergillus* sp. and the length of the clinical course of aspergillosis. The findings suggested that pathological evidence was not associated with the cluster analysis and patients with different pathological conditions (e.g., IPA, CPA, and ABPA) could be allocated to the same group. In this study, the result of cluster analysis might be explained by: (1) the first group (CCPA,CFPA) had the same infectious severity that both had a longer length of the clinical course (>6 months); (2) the second group might all be involved with the distribution of fungal agents within the respiratory airways and similar EBC metabolites were found. Due to its invasive nature, IPA and CNPA could cause invasion of the respiratory tract. Aspergillus nodule might be caused by the distribution of *Aspergillus* sp. along the respiratory tract. ABPA is a type of reactive airway diseases; and (3) aspergilloma usually presents with no or mild symptoms, or no radical changes within at least 3 months. Due to mild symptoms, patients usually resolve without any treatment. Unfortunately, if CCPA presents in a single cavity, it is difficult to discriminate it from aspergilloma, and further information, such as clinical symptoms, progression revealed by radiological imaging, and inflammatory biomarkers are required for the discrimination. However, the findings suggested a potential diagnostic tool for discriminating between chronic cavitary PA and aspergilloma. This was because some compounds are associated with the severity of *Aspergillus* sp. infection.

For the diagnosis of PA (IPA, CPA, and ABPA), the panels of metabolites in the EBC samples have several advantages. First, the EBC sample was easy to prepare and could be stored and transferred for analysis. Most importantly, this method is noninvasive. Second, the control group included pneumonia and chronic respiratory tract infections, such as *Pseudomonas aeruginosa* and *Stenotrophomonas maltophilia* colonization. the EBC method could easily differentiate them. This implied that the metabolites found in this study were specific to PA patients. Third, the sensitivity of routine methods appeared to decrease during treatment, only 20% were positive using routine methods after 6 months of therapy and none were positive using routine methods when patients were censored. Unlike routine methods, the sensitivity of the EBC method remained high and was always 100% during the treatment. In addition, even in patients that were free of abnormal radiological features, ≥1 metabolite in the EBC samples could be detected. Similar finding could be found in *Aspergillus* colonization controls. These finding has equal significance with a positive *Aspergillus* PCR in BALF (Denning et al., 2015), which supports the diagnosis, but are not enough alone for a confirmed diagnosis of PA as numerous other conditions can yield *Aspergillus* in the airways. However, after antifungal therapy termination, the target metabolites couldn’t be detected. This study has several limitations. First, due to the superior sensitivity of mass spectrometry, levels of metabolites in the EBC samples varied significantly between each run. Therefore, this study was conducted based on qualitative data. In addition, the level of metabolites found in the study was not further analyzed and investigated for their potential significance. Second, for *Aspergillus* sp. infection, several clinical presentations, such as invasive PA, allergic bronchopulmonary aspergillosis, and chronic PA might occur in one patient. This might contribute to a significant influence on the accuracy of cluster analysis. Third, Aspergillus-specific IgG antibodies serology test, which is known as a superior test to other diagnostic methods for CPA confirmation, was infrequently used in the cohort. Therefore, a comparison with EBC method wasn’t evaluated, this point should be paid an caution. Fourth, small sample is another limitation. For example, due to few cases of IPA and ABPA included, our results may not reflect the true metabolic profile of EBC samples. If more controls with *Aspergillus* colonization were included, metabolite for the discrimination between *Aspergillus* colonization and diseases may be identified. In the next, further analysis is required to validate the findings in a larger population.

This study investigated the EBC metabolic profile of PA patients using UHPLC/ESI–HRMS and five biomarkers for the diagnosis of PA were identified. Compared with other methods (e.g., sputum smear and culture, BALF GM assay, and mNGS), the EBC method was accurate and efficient. More importantly, the EBC method remained positive for a long time, up to 1 year later. In addition, the metabolite profiling of EBC samples could reveal the metabolite signatures in patients with different clinical presentations of PA. These findings suggested that the EBC method could be a potentially safe, noninvasive approach for the diagnosis of patients with PA.

## Data availability statement

The original contributions presented in the study are included in the article/**Supplementary Material**. Further inquiries can be directed to the corresponding authors.

## Ethics statement

The study was conducted at Fujian Provincial Hospital in Southeast China. This study was approved by the Ethics Committees of Fujian Provincial Hospital. Written informed consent was obtained from all suspected patients before the study started.

## Author contributions

SW contributed to the study design, data collection, data analysis, data interpretation, funding acquisition, and figures and wrote the manuscript. Y-sC contributed to the data curation, data analysis, data interpretation, and figures. YS contributed to the conceptualization and design of the study. SW and Y-sC contributed equally to this work and should be considered co-first authors. SW is the first corresponding author. YS is the co-corresponding authors. All authors contributed to the article and approved the submitted version.

## Funding

This study was funded by the Natural Science Foundation of Fujian Province (2021J01377).

## Conflict of interest

The authors declare that the research was conducted in the absence of any commercial or financial relationships that could be construed as a potential conflict of interest.

## Publisher’s note

All claims expressed in this article are solely those of the authors and do not necessarily represent those of their affiliated organizations, or those of the publisher, the editors and the reviewers. Any product that may be evaluated in this article, or claim that may be made by its manufacturer, is not guaranteed or endorsed by the publisher.
