# Supplementary material for: Metabolomic profiling of exhaled breath condensate for the diagnosis of pulmonary aspergillosis
Source: Front Cell Infect Microbiol. 2022 Sep 8;12:1008924. doi: 10.3389/fcimb.2022.1008924 (PMC9492867; doi:10.3389/fcimb.2022.1008924)
Supplement: Supplementary file 1 [file Presentation_1.pdf]

UHPLC-HRMS parameters

(1) Liquid chromatography parameter settings: Ultra performance liquid chromatography (UPLC) setup in Waters Xeno G2-XS QTOF with the following separation conditions: ethylene-bridged hybrid (BEH) C18 column (column 2.1 mm x 150 mm, 1.7μm); methanol and ultrapure water gradient elution. Methanol: ultrapure water (1:9) gradient elution for 2 min at the beginning, followed by methanol: ultrapure water (9:1) rinse for 2 min, and finally methanol: ultrapure water (1:9) gradient elution for 1 min; flow rate, 0.6 mL/min; column temperature at 35℃, sample temperature at 15°C; injection volume, 3μL. 5 min for each sample gradient elution, followed by column wash for 5 min.

(2) Mass spectrometry parameter settings: Positive ion MS ES+ mode was selected for the analysis setup, and the mass spectra were acquired in the mass-to-charge ratio range of 100-1000Da. The following parameters were set: capillary voltage (kV): 2.5-3.0kV; sample cone voltage: 30-60V; extraction cone voltage: 4V; source temperature: 100℃; desolvent temperature: 300-400℃; cone gas flow rate: 50L/h; desolvent gas flow rate: 600-800L/h. Mass-to-charge ratio range: 100-1000Da
